# Effect of connector size and design on the fracture resistance of monolithic zirconia fixed dental prosthesis

**DOI:** 10.34172/joddd.2020.039

**Published:** 2020-11-09

**Authors:** Ali Hafezeqoran, Roodabeh Koodaryan, Yasser Hemmati, Ayshin Akbarzadeh

**Affiliations:** ^1^ Department of Prosthodontics, Dental faculty, Tabriz University of Medical Sciences, Tabriz, Iran

**Keywords:** Dental ceramics, Fixed partial denture, Load-bearing, Monolithic zirconia

## Abstract

**Background.** Designing a high strength all-ceramic fixed partial denture with favorable esthetics can be challenging for clinicians; this study aimed to evaluate the effect of connector size and design on the fracture resistance of monolithic zirconia fixed dental prostheses.

**Methods.** Two groups of twenty 3-unit monolithic zirconia (Sirona inCoris TZI, Sirona Dental Systems GmbH) bridges, extending from the mandibular first premolar to the first molar with different connector sizes (9 mm^2^ and 12 mm^2^), were divided into two subgroups with different connector designs (round and sharp). The specimens were subjected to the three-point bending test to obtain the fracture-bearing load. The results were reported using descriptive statistics (mean ± standard deviation). Mann-Whitney U test was used to compare the fracture load in two types of designs for each connector size and two connector size types for each connector design. The significance level was considered at *P*<0.05.

**Results.** The minimum failure load was related to the group with a 9-mm^2^ connector size and a sharp embrasure design (1054.4±133.89 N), and the highest mean value belonged to the group with 12-mm^2^ connector size and rounded embrasure design (1599.8±167.09 N). Mann-Whitney U test indicated a significant difference between the mean failure load of the rounded and sharp embrasure designs in the 9-mm^2^ connector size (*P* =0.007). However, the difference was insignificant in the 12-mm^2^ connector size (*P* =0.075).

**Conclusion.** Sharp embrasure design is not recommended for high-stress areas with restricted occlusogingival height. A 9-mm2 connector size for 3-unit monolithic zirconia fixed dental prosthesis (FDP), which is recommended by the manufacturer, should be used more cautiously

## Introduction

Metal, ceramic, and porcelain-fused-to-metal prostheses are considered available materials to fabricate fixed dental prostheses (FDPs). In this regard, ceramics have attracted more attention, especially for esthetic zone restorations in recent years,^1^ leading to the introduction of various types of dental ceramics with satisfactory esthetic and biocompatibility.^2^ However, the application of ceramics was first limited to single crowns or anterior short-span FDPs due to their low mechanical properties.^3^ Yttrium-stabilized tetragonal zirconia (Y-TZP) has been selected as the material of choice in high-stress regions due to its high strength, biocompatibility, and acceptable esthetic.^4^

Several studies have confirmed the use of zirconia as a core material for long-span FDPs in high-stress clinical situations.^5^ Compared to metal-ceramic restorations, zirconia has a better aesthetic appearance; however, layering with more translucent materials is essential for enhancing the restoration’s appearance concerning its opacity.^6^ Unfortunately, clinical results about zirconia-based crowns or FDPs are associated with several short-term failures mainly due to cohesive fracture in the porcelain layering the core despite its superior mechanical properties.^7^ This value is significantly higher compared to the delayering of porcelain-fused-to-metal restorations.^8^ Agustín-Panadero et al^6^ reported a 6%‒15% fracture rate for porcelain covering the zirconia core in FPDs during a 3‒5-year period, with 4% within 10 years for metal-ceramic restorations.

The tendency to eliminate the problem of porcelain chipping in Y-TZP restorations led to the introduction of full-contour zirconia restorations.^9^ Monolithic nature of these restorations resolves the problem related to veneer chipping and while an increase in the Y-TZP thickness enhances the FDP strength.^10^ Producing a relatively translucent zirconium is regarded as the initial requirement for producing monolithic zirconia restorations, which is obtained by applying some structural changes and altering the sintering process; however, the mechanical outcome is unclear.^5^ According to Malkondu et al,^9^ monolithic zirconia presents a significant strength even in low thicknesses. A study by Sulaiman et al^11^ showed a higher fracture rate in FDPs than single crowns; however, the overall failure value was low in five years.^11^

The connector area should be constricted for aesthetic and biologic reasons^12^; however, it is recommended to make the connector area as wide as possible to assure the maximum clinical performance of ceramic FDPs.^13^ The minimum recommended dimension for connectors varies from 2 to 4 mm in different studies^4^; therefore, designing a high-strength ceramic FDP with favorable esthetics can be regarded as a challenge for the clinician.^14^ In stressful areas, like posterior FDPs with decreased occlusal height, altering the connector design might play an important role in increasing the fracture load.^12^ Hamza et al^4^ concluded that rounded embrasure design bears higher occlusal loads than the sharp design when connector dimensions decrease.

Several factors can alter the load-bearing capacity of an all-ceramic prosthesis. The present study aimed to evaluate the effect of connector size and design on the fracture resistance of monolithic zirconia fixed dental prostheses. The study’s null hypothesis stated that connector size and design has no effects on the fracture resistance of monolithic zirconia fixed dental prosthesis.

## Methods

Forty 3-unit posterior FDPs, extended from the mandibular first premolar to the first molar, were milled from translucent pre-dyed partially sintered zirconia ceramic discs (Sirona inCoris TZI, Sirona Dental Systems GmbH). The specimens were divided into two main groups based on the connectors’ surface area, and each group was classified into two subgroups based on the embrasure radius of the curvature.

### Preparing the specimen

**Figure 1:**
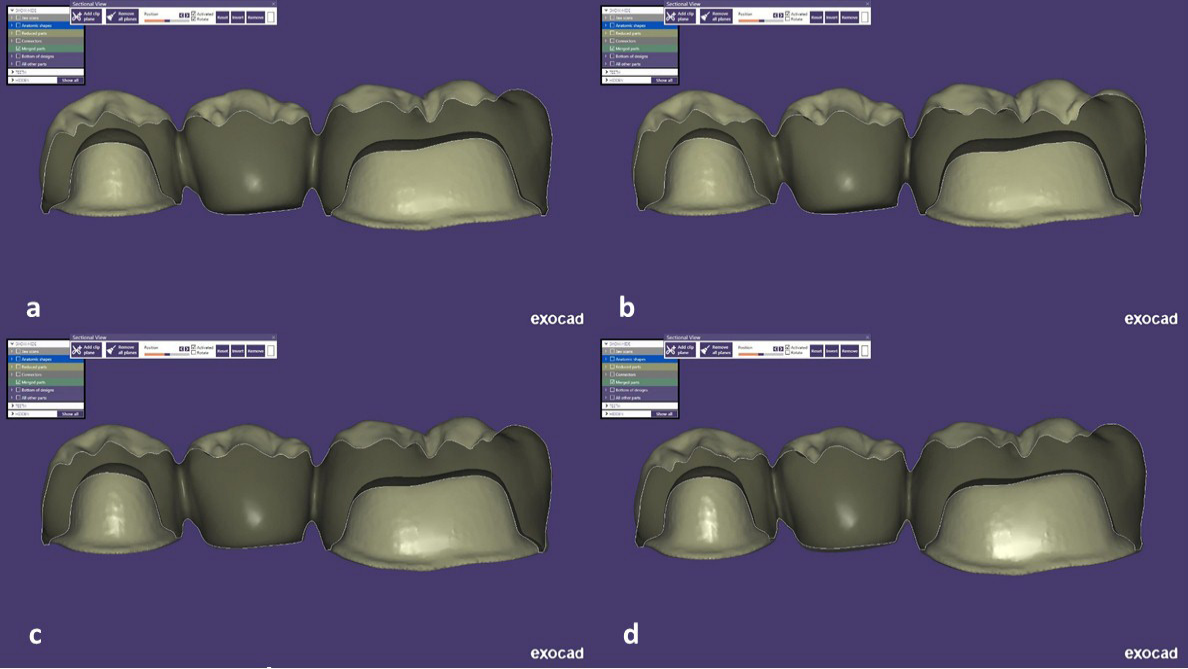
A cross-sectional view of designed FDPs; a. group A; b. group B; c. group C; and d. group D.

The abutment teeth (mandibular first premolar and first molar) were prepared on a standard plastic jaw model (prosthetics restoration jaw model PRO 2001-UL-HD-M-32, Nissin Dental Product Inc., Kyoto, Japan) by considering some parameters, like 6 mm of occlusal height, chamfer finish line with an 0.5-mm depth, and a 6° convergence angle.^15,16^ Then, all the transitions from the axial to the occlusal or incisal areas were rounded off, and a mesiodistal groove was simulated on the occlusal surface. Accordingly, an impression was made from the master model with additional silicon (Panasil, Kettenbach, Germany) and poured with die stone (Kerr Vel-Mix Stone ISO type IV, Kerr Europe AG, Basel, Switzerland) to make stone replicas of the master model for the scanning process.^17^ Each stone model was scanned with a laboratory scanner (inEOS X5, Sirona Dental Systems, Bensheim, Germany), and FDPs were designed using CAD software (Exocad, Exocad GmbH, Darmstadt, Germany).^16^ The restorations were 1-mm thick in the central groove and 0.8-mm thick at axial walls. Additionally, the cement gap was set at 45 µm, and the buccolingual width of the pontics was 8 mm. Four connector types were designed for four study groups (Figure 1) as follows:

**Group A:** Ten 3-unit FDPs with 9-mm^2^ connector size and rounded embrasure design with an 0.9-mm curvature radius.

**Group B:** Ten 3-unit FDP with 9-mm^2^ connector size and sharp embrasure design with an 0.25-mm curvature radius.

**Group C:** Ten 3-unit FDP with 12-mm^2^ connector size and rounded embrasure design with an 0.9-mm curvature radius.

**Group D:** Ten 3-unit FDP with 9-mm^2^ connector size and sharp embrasure design with an 0.25-mm curvature radius.

In the next stage, the designed FDPs were milled with a CAM unit (Cerec inLab; Sirona Dental Systems, Bensheim, Germany). After the milling process, all the specimens separated from the disks and the remaining specimen were cleaned with compressed air according to the manufacturer’s recommendation to prevent any milling residues. Then, the restorations were sintered in a proper furnace (Vita Zyrcomat T, Vita, Germany) following the manufacturer’s instructions. All the milled restorations were polished properly and checked for marginal integrity with condensational silicon light body (Fit Checker, GC, Tokyo, Japan) on stone models.^13^

### Preparing the test models

The impressions were made from stone models with additional silicon and poured with epoxy resin to fabricate a final model for the testing procedure. Then, each specimen was cemented to one resin model with resin cement (Panavia F 2.0, Kuraray, Kurashiki, Okayama, Japan) following the manufacturer’s instructions. All the cemented restorations were immersed in distilled water at 37°C for 24 hours before performing the test.^13^

### Measuring the fracture resistance

All the specimens were exposed to a three-point bending test using a universal test machine (HOUNSFIELD H25KS, Hounsfield, UK). They were vertically loaded with a 6-mm steel ball at a crosshead speed of 1 mm/min at the pontic center.^3^ The maximum load was recorded after a sudden decrease in the force, as a fracture load.

### Statistical analysis

Kolmogorov-Smirnov test was used for analyzing the normal distribution of the data. Mann-Whitney *U* test was used separately in each design to compare the fracture load in two dimensions of the connector. The same tests were used to compare the fracture load in rounded and sharp designs separately for each connector dimension. Statistical analysis was performed using SPSS 17 at the significance level of *P*< 0.05.

## Results

Table 1 presents the descriptive statistics related to the failure load. The minimum failure load was recorded in group B (1054.4±133.89 N), and the highest mean value was recorded in group C (1599.8±167.09 N). As shown, the mean failure load in both connector sizes was higher in the round embrasure design compared to the sharp one. In both embrasure designs, the mean failure load in the 9-mm^2^ connector size was lower than the groups with 12-mm^2^ connector size. In addition, the minimum and maximum failure loads were recorded in group B (878 N) and C (1974 N), respectively.

**Table 1:** Descriptive statistics

**Dimension**	**Design**	**Mean ± SD**	**Minimum**	**Maximum**
9 mm^2^	Round	1327.4±196.37	936	1587
	Sharp	1054.4±196.37	878	1250
12 mm^2^	Round	1599.8±167.09	1380	1974
	Sharp	1440±159.05	1093	1672

Kolmogorov-Smirnov test was used to analyze the normal distribution of data before comparing the failure load in two embrasure designs and two different connector dimensions. Based on the results, the data were not normally distributed for the failure load values (*P* < 0.05). Therefore, the Mann-Whitney U test, as a nonparametric test, was used for the comparisons. As shown in Table 2, the results of the Mann-Whitney *U* test indicated a significant difference between the mean failure load of the rounded and sharp embrasure designs in the 9-mm^2^ connector size (*P* = 0.007). However, the difference was not significant in the 12-mm^2^ connector size (*P* = 0.075). Regarding the rounded embrasure design, the difference between the mean failure load in two connector sizes was statistically significant (*P* = 0.002). Additionally, the difference was significant for the sharp embrasure design (*P* < 0.001).

**Table 2:** The comparison of failure load in different dimensions and designs

**Connector**	**MD**	***P*** ** value**
Rounded‒sharp (9 mm^2^)	273	0.007
Rounded‒sharp (12 mm^2^)	159.8	0.075
9‒12 mm^2^ (round)	-272.4	0.002
9‒12 mm^2^ (sharp)	-385.6	<0.001

MD: mean deviation.

## Discussion

Increasing aesthetic demands have prompted dentists to use all-ceramic prostheses to replace missing teeth; in this regard, zirconia is one of the strongest aesthetic materials available. The present study investigated the effect of connector size and design on the fracture resistance of monolithic zirconia FDPs. According to the results, changes in the size and design of monolithic zirconia FDPs altered fracture resistance; therefore, the study’s null hypostasis was rejected.

In the present study, attempts were made to reproduce clinical conditions. This study’s specimens were designed as 3-unit FDPs with normal anatomic contours instead of bar-like specimens used in previous studies,^4,15^ which were unrelated to a fixed partial denture. In addition, epoxy resin was used as the supporting structure of the specimen, as its elastic modulus is placed between the elastic modulus of cancellous bone and dentin.^12^

Based on the present study results, an increase in the connector surface area has a significant effect on the load-bearing capacity of monolithic zirconia FDP. The mean failure loads in 12-mm^2^ connector size were approximately 136% and 120% higher than the 9-mm^2^ in sharp and rounded embrasure design. The results are consistent with previous studies on zirconia and other ceramic core material.^3,4,7,12^ However, no study was found on the fracture resistance of monolithic zirconia FDPs according to the authors’ search.

The fracture resistance of an FDP should be high enough to bear the patient’s maximum bite force, which depends on the patient’s age, gender, dental status, and the like.^18,19^ The value varies from 216 to 847 in the posterior region,^20^ while the load-bearing threshold should be at least 500 N for posterior FDPs.^3^ Considering the repeated occlusal forces applied to FDPs in clinical conditions, fatigue fracture can play a major role in restoration survival. It is recommended that the fracture resistance of ceramics should be >1000 N for a better clinical performance as a posterior FDP^12,20^ since the endurance limit for ceramics is 40%‒50% of their ultimate strength.

According to the manufacturer, the appropriate connector area for a 3-unit posterior FDP is 9 mm^2^. However, regarding the present study results, the mean failure load value was >1000 N in all the groups, while it was <1000 N in some of the specimens in groups A and B. The minimum failure load was recorded in the sharp and rounded embrasure design groups (878 N and 936 N), indicating the failure possibility in the intraoral environment and under repetitive forces. Therefore, it is recommended that 9-mm^2^ connectors be used with more caution despite the manufacturer’s recommendation.

A statistically significant difference was reported between sharp and rounded embrasure designs in specimens with a 9-mm^2^ connector area. The result can help the clinician choose a rounded connector design in posterior FDPs with restricted occlusal space to enhance the restoration’s fracture resistance and clinical performance to an acceptable value.

The different embrasure designs in the groups with the 12-mm^2^ connector area resulted in no significant failure load difference, different from the results of previous studies.^3,4,7,12^ Thus, the laboratory technician can have the opportunity to design the connector area without considerable changes in restoration strength in the areas with sufficient occlusogingival height.

In the present in vitro research, some limitations might be related to differences between in vivo and in vitro environments, possibly affecting the results. The differences in the structure and elastic modulus of supporting units, intraoral aqueous conditions, load directions, and intensity variations related to specimens, compared to static vertical loads in laboratory studies, are considered as some examples. However, in vivo studies are more time-consuming and expensive, and it is not easy to control the confounding variables in such studies. Furthermore, it is better to evaluate the results of the current study with caution by considering each case’s unique needs. The intraoral conditions, such as thermal changes and dynamic intermittent loads, were not simulated in this study, which might have affected the restoration’s performance. Therefore, more in vivo and long-term studies should be conducted.

## Conclusion

In the present study, the following conclusions were drawn:

- Increasing connector dimensions in monolithic zirconia FDPs increases fracture resistance. Second, a sharp embrasure design is not recommended for high-stress areas with restricted occlusogingival height.
- A 9-mm^2^ connector dimension for a three-unit monolithic zirconia FDP,which is recommended by the manufacturer, should be used more cautiously.
- A 12-mm^2^ connector size is strong enough for a three-unit monolithic zirconia FDP, irrespective of embrasure design.

## Authors’ Contributions

AH: concept, design, manuscript review; RK: data analysis, statistical analysis, manuscript editing; YH: literature search, data acquisition, manuscript preparation; AA: definition of intellectual content, literature search, experimental studies, data acquisition, manuscript preparation.

## Acknowledgments

The authors would like to appreciate the Vice Chancellor for Research, Tabriz University of Medical Sciences, for the financial support of this research project.

## Funding

The study was financially supported by the Vice Chancellor for Research, Tabriz University of Medical Sciences.

## Competing Interests

The authors declare no competing interests with regards to the authorship and publication of this article.

## Ethics Approval

The study protocol was approved by the Ethics Committee under the code IR.TBZMED.VCR.REC.1397.414.

## References

[1] Taskonak B, Yan J, Mecholsky JJ Jr, Sertgöz A, Koçak A (2008). Fractographic analyses of zirconia-based fixed partial dentures. Dent Mater.

[2] Raigrodski AJ, Hillstead MB, Meng GK, Chung KH (2012). Survival and complications of zirconia-based fixed dental prostheses: a systematic review. J Prosthet Dent.

[3] Kohorst P, Herzog TJ, Borchers L, Stiesch-Scholz M (2007). Load-bearing capacity of all-ceramic posterior four-unit fixed partial dentures with different zirconia frameworks. Eur J Oral Sci.

[4] Hamza TA, Attia MA, El-Hossary MM, Mosleh IE, Shokry TE, Wee AG (2016). Flexural strength of small connector designs of zirconia-based partial fixed dental prostheses. J Prosthet Dent.

[5] Stober T, Bermejo JL, Rammelsberg P, Schmitter M (2014). Enamel wear caused by monolithic zirconia crowns after 6 months of clinical use. J Oral Rehabil.

[6] Agustín-Panadero R, Román-Rodríguez JL, Ferreiroa A, Solá-Ruíz MF, Fons-Font A (2014). Zirconia in fixed prosthesis A literature review. J Clin Exp Dent.

[7] Koenig V, Vanheusden AJ, Le Goff SO, Mainjot AK (2013). Clinical risk factors related to failures with zirconia-based restorations: an up to 9-year retrospective study. J Dent.

[8] Chang JS, Ji W, Choi CH, Kim S (2015). Catastrophic failure of a monolithic zirconia prosthesis. J Prosthet Dent.

[9] Malkondu Ö, Tinastepe N, Akan E, Kazazoğlu E (2016). An overview of monolithic zirconia in dentistry. Biotechnol Biotechnol Equip.

[10] Abdulmajeed AA, Lim KG, Närhi TO, Cooper LF (2016). Complete-arch implant-supported monolithic zirconia fixed dental prostheses: A systematic review. J Prosthet Dent.

[11] Sulaiman TA, Abdulmajeed AA, Donovan TE, Cooper LF, Walter R (2016). Fracture rate of monolithic zirconia restorations up to 5 years: a dental laboratory survey. J Prosthet Dent.

[12] Oh WS, Anusavice KJ (2002). Effect of connector design on the fracture resistance of all-ceramic fixed partial dentures. J Prosthet Dent.

[13] Onodera K, Sato T, Nomoto S, Miho O, Yotsuya M (2011). Effect of connector design on fracture resistance of zirconia all-ceramic fixed partial dentures. Bull Tokyo Dent Coll.

[14] Lakshmi RD, Abraham A, Sekar V, Hariharan A (2015). Influence of connector dimensions on the stress distribution of monolithic zirconia and lithium-di-silicate inlay retained fixed dental prostheses–A 3D finite element analysis. Tanta Dent J.

[15] Plengsombut K, Brewer JD, Monaco EA Jr, Davis EL (2009). Effect of two connector designs on the fracture resistance of all-ceramic core materials for fixed dental prostheses. J Prosthet Dent.

[16] Villefort RF, Amaral M, Pereira GK, Campos TM, Zhang Y, Bottino MA (2017). Effects of two grading techniques of zirconia material on the fatigue limit of full-contour 3-unit fixed dental prostheses. Dent Mater.

[17] Sundh A, Molin M, Sjögren G (2005). Fracture resistance of yttrium oxide partially-stabilized zirconia all-ceramic bridges after veneering and mechanical fatigue testing. Dent Mater.

[18] Bahat Z, Mahmood DJ, Vult von Steyern P (2009). Fracture strength of three-unit fixed partial denture cores (Y-TZP) with different connector dimension and design. Swed Dent J.

[19] Koc D, Dogan A, Bek B (2010). Bite force and influential factors on bite force measurements: a literature review. Eur J Dent.

[20] Tinschert J, Natt G, Mautsch W, Augthun M, Spiekermann H (2001). Fracture resistance of lithium disilicate-, alumina-, and zirconia-based three-unit fixed partial dentures: a laboratory study. Int J Prosthodont.

